# Complications of Paediatric Flexible Bronchoscopy with Six-Lobe Bronchoalveolar Lavage Performed Under General Anaesthesia †

**DOI:** 10.3390/pediatric18020031

**Published:** 2026-02-26

**Authors:** Maria van Veelen, Kelly Bakewell, Christopher W. A. Jolley, Sheng-Ang Ho, James Chapman, Lauren Edwards, Rahul Kumar, Francis J. Gilchrist

**Affiliations:** 1 Department of Paediatric Respiratory Medicine, Staffordshire Children’s Hospital at Royal Stoke, University Hospitals of North Midlands NHS Trust, Stoke-on-Trent ST4 6QG, UKfrancis.gilchrist@uhnm.nhs.uk (F.J.G.); 2 Department of Paediatric Anaesthesia, Staffordshire Children’s Hospital at Royal Stoke, University Hospitals of North Midlands NHS Trust, Stoke-on-Trent ST4 6QG, UK; 3 Faculty of Medicine and Health Sciences, Keele University, Keele ST5 5BG, UK

**Keywords:** paediatric, respiratory, bronchoscopy, bronchoalveolar lavage, complications

## Abstract

Aim: To undertake a prospective review to identify the intra-procedure complications in children undergoing flexible bronchoscopy with six-lobe lavage and a retrospective review to identify the rates of delayed discharge and readmission. Methods: The prospective review analysed consecutive procedures from August 2023 to August 2024 and collected data on intra-procedure and immediate post-procedure desaturations, laryngospasm, bronchospasm/wheeze, tachypnoea, pyrexia, hypothermia, and vomiting. The retrospective review analysed consecutive paediatric flexible bronchoscopies from October 2014 to August 2023 identifying discharge delays and readmissions. All children underwent flexible bronchoscopy at a single tertiary paediatric centre under general anaesthesia (GA) with a single aliquot BAL obtained from all six lobes. When cytology was required, the BAL from the right middle or most affected lobe was changed to triple aliquot. Results: Six hundred and twenty-two procedures performed on 540 children were analysed. This included 502 in the retrospective review and 120 in the prospective review. In the prospective group 4/120 (3.3%) children experienced a significant (<90%) desaturation requiring anaesthetic intervention; 11/120 (9.2%) experienced an immediate post-procedure complication such as desaturation, pyrexia, tachypnoea, wheeze, or vomiting; 53/622 (8.5%) had their discharge delayed overnight; and 13/120 (11%) children in the prospective group experienced hypothermia. A further 18/622 (3%) children re-attended hospital within 48 h of discharge. Conclusions: Flexible bronchoscopy with bronchoalveolar lavage in all six lobes under GA in children is a safe procedure with low incidence of major complications when performed by expert clinicians. Parents should be advised of a 9% risk of delayed overnight discharge.

## 1. Introduction

It has now been over 40 years since the first flexible bronchoscopies (FB) were performed on children [1]. Applications of paediatric bronchoscopy have expanded during this period to meet medical and technological advancements. Guidelines in respect of the methodology have been published, but there remains variation in practice regarding bronchoalveolar lavage (BAL) samples [2,3,4,5]. The European Respiratory Society (ERS)’s guidance on BAL in children suggests a single BAL specimen should be taken from the most affected lobe or the right middle lobe [2], and this is echoed by the American Thoracic Society (ATS) [3]. Subsequent observational studies have shown an increased microbiological yield in children with cystic fibrosis (CF) and protracted bacterial bronchitis (PBB) when six lobes (including lingula) are sampled [5,6,7,8].

Another notable component of paediatric respiratory procedures lacking in the literature is the optimal room temperature. Although theatre temperature is well documented for burns and neonates, there is a paucity of evidence regarding recommended room temperature for paediatric bronchoscopies to prevent hypothermia. Although most bronchoscopies are short in duration, paediatric patients are still at risk of heat loss due to their body surface area, even patients that are past infancy [9,10]. In the United Kingdom (UK), there are currently no clear guidelines for the minimum room temperature for paediatric patients, and thus many hospital trusts have their procedural rooms and theatres set to the adult minimum standard of 21° Celsius [11].

At our tertiary paediatric respiratory centre, we perform 120–140 paediatric bronchoscopies per year. If the primary indication is to obtain microbiological samples, our standard practice is to obtain single aliquot BALs from six lobes (including lingula). We are aware that other centres have been reluctant to adopt this practice due to a potential increase in side effects. We therefore wanted to report on this as the only previous large study presenting the complications of paediatric flexible bronchoscopy performed their procedures under light or deep sedation, not general anaesthesia (GA), with only one to two lobes lavaged on each child [12].

## 2. Materials and Methods

A prospective review was performed on all paediatric patients undergoing FB-BAL with 6-lobe lavage at our centre from August 2023 to August 2024. We measured their rates of intra-procedural and immediate post-procedural complications, as well as the rates of hypothermia. We also undertook a retrospective case note review of all paediatric patients who had undergone flexible bronchoscopy from October 2014 to August 2023 collecting data on delayed discharges and re-admission rates.

**Figure 1:**
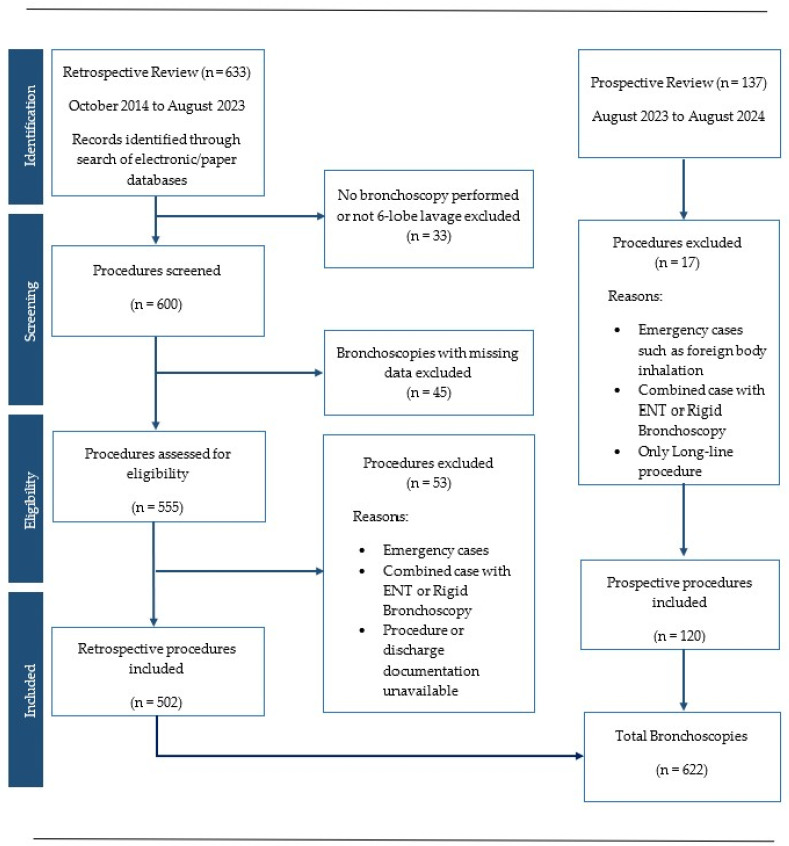
Retrospective and prospective case note analysis with initial identification of cases, screening of appropriate bronchoscopies, eligibility for inclusion, and final procedures included.

All children at our tertiary paediatric centre under the age of 18 who underwent FB-BAL with six-lobe lavage were included (Figure 1). Cystic fibrosis patients with multiple bronchoscopies over the 9-year period from October 2014 to August 2023 were included (Figure 1). Emergency cases, rigid bronchoscopies, and combined procedures with the Ear, Nose & Throat (ENT) surgeons were excluded (Figure 1).

The following were defined as major complications: significant desaturation (${SpO}_{2}$ ≤ 90%), intra-procedural laryngospasm, intra-procedural bronchospasm, wheeze, intra-procedural coughing, and pneumothorax. The following were defined as minor complications: transient desaturation (${SpO}_{2}$ 91–94%) and immediate post-procedural cough, pyrexia, tachypnoea, vomiting, or wheeze. Although transient periods of desaturation below 90% could be considered a minor complication, all intra-procedural and immediate post-procedural desaturations equal to or below 90% were categorised as a major complication in accordance with previously established definitions [2,3,4,12].

Our FBs are carried out in a procedural room with a paediatric anaesthetic consultant, paediatric respiratory consultant, operating department practitioner (ODP), and paediatric respiratory advanced nurse practitioner (ANP) always present. Each child is continuously monitored with three-lead electrocardiography, pulse oximetry (arterial oxygen saturation), and end-tidal carbon dioxide. A full set of observations are also taken within one hour pre-operatively, immediately post-procedure, every 15 min post-operatively for one hour, then every hour for the following three hours if the child remains stable. For the hypothermia portion of our study, pre-operative baseline temperatures and immediate post-operative temperatures were taken using tympanic thermometers, which were calibrated weekly. The temperature of the procedural room was measured through our tertiary centre’s engineering department, which was provided as a 30 min read-out of room temperatures daily.

For our procedures, the anaesthetists use sevoflurane carried with a mixture of air and oxygen. If there is a concern regarding reactive airways, intravenous propofol may be used. The bronchoscope is introduced to the airway through a laryngeal mask after the anaesthetist has sprayed the larynx and subglottic area with 1% lidocaine using a mucosal atomisation device (MAD). Single aliquot BALs are obtained from all six lobes, including the lingula. The tip of the scope is wedged into a lobar bronchus and a 0.9% saline lavage of 0.5–1 mL/kg, with a maximum volume of 20 mL, is instilled and retrieved using a syringe attached to the bronchoscope channel.

In patients with diffuse disease, the lobes are sampled in a set order from right upper, right middle, right lower, left upper, lingula, to left lower. In patients with an identified most affected lobe, this is sampled first, and then the previous order is followed. BAL samples are sent to the microbiology laboratory for semiquantitative bacterial cultures, and visible growth is categorised. If a cytology sample is required, a triple aliquot BAL is performed in the most affected lobe or the right middle lobe. The first aliquot is sent to the microbiology laboratory for semiquantitative bacterial cultures. The second and third aliquots from triple aliquot BALs are mixed and sent for cytology.

## 3. Results

The prospective review conducted from August 2023 to August 2024 had a final sample size of 120 (Figure 1). The retrospective case review looking at procedures from October 2014 to August 2023 had 502 cases, for a combined evaluation of 622 bronchoscopies (Figure 1). The median (SD) age at FB-BAL was 3.5 (3.0) years old (range: 3 months–17 years). The commonest indications for FB-BAL were PBB and CF (Table 1).

The vast majority of children (87.5%) in the prospective review did not experience any complications (Table 2). Four children (3.3%) had a significant complication with desaturations to <90%. In one, this was due to laryngospasm, and in another to a cuff leak (Table 3). Further details of these four complications are given in Table 3.

Discharge was delayed in 60 (9.6%) out of 622 children (Table 4). In seven (1.1%) this caused a late evening discharge and in 53 (8.5%) it resulted in an unplanned overnight stay. The reasons for these delayed discharges are shown in Table 4. Eighteen (3.3%) patients reattended the children’s assessment unit (CAU) or the paediatric ambulance and emergency (A&E) department within 48 h of their bronchoscopy. The reasons for this are shown in Table 5.

The mean procedure duration in our prospective group was 33 minutes (range: 20–81 min). The baseline pre-operative tympanic temperature mean was 36.6 °C (range: 35.7–37.6 °C) and the immediate post-operative mean was 37.0 °C (range: 35.5–37.3 °C). Thirteen (10.8%) of our children in the prospective group had hypothermia (<36 °C), with one child measuring below 36 °C in the pre-operative period. The temperature of 81 (67.5%) of the 120 children in the prospective group dropped during their procedure. The mean fall in temperature was 0.5 °C. The average procedure room temperature was 22.8 °C. Our bronchoscopy room has no windows, a single door, and is measured at 57 $m^{3}$.

## 4. Discussion

To our knowledge, this is the first published review of complications associated with paediatric FB with six-lobe BAL under GA. The results indicate this procedure is well tolerated with a low risk of major complications (<4%). An important element of our study that adds to clinical relevance is that it includes a significant proportion of children with hyperreactive airways and heightened risks from GA. Of the 128 children with CF (20.6%), 82 had multiple bronchoscopies over the 10-year period. Our children with CF, difficult asthma, non-CF bronchiectasis, and primary ciliary dyskinesia (PCD) have increased risks of complications during GA, yet none of them experienced major complications. This may be partly attributed to careful planning of elective admissions by the paediatric respiratory team, and/or more stringent preparedness from the paediatric anaesthetist.

Previously published data on paediatric FB has demonstrated a risk of <2% for major complications and 5.2% for minor complications [12]. Although de Blic, Marchac, and Scheinmann had a much larger prospective sample size, only 7.2% of their procedures were performed under deep sedation, with all others performed on conscious patients under light sedation [12]. Owing to this fact and the added risks from GA, we feel that sampling all six lobes including the lingula does not greatly contribute to the incidence of major complications. Of the 11 (9.2%) minor complications, transient desaturation predominated (6 cases), which supports the previous findings that this occurs more frequently in procedures operating deeper sedation [12]. Our delayed overnight discharge rate was 8.5% and re-admissions were 3%, but there has been no antecedent published data that has measured either the delayed day case procedure discharges caused by major or minor complications, and the rates of re-admissions following paediatric FBs.

However, complications are not preventable, and hypoxia remains the greatest risk of FB- BAL under GA. Given that our day case unit has more strict guidelines of providing facemask oxygen when the post-operative saturation falls below 95%, children are more likely to be observed longer in the evening or overnight than in other centres which might use 92% as a cut-off value. Overall, post-bronchoscopy fever remained the most frequent minor complication, accounting for 60% of delayed discharges, and 89% of re-admissions. A greater proportion of children who may have developed a fever following discharge might not be accounted for as they did not re-present at our unit.

The complications of FB-BAL under GA in children are not well documented. Despite one large prospective study and several task force committee hearings, clear distinctions between major and minor complications during and immediately after FB-BAL in children do not have strict definitions in the literature [2,3,4,12]. Given the lack of comparative studies, the previously defined threshold for an oxygen saturation of below 90% was utilised, despite the additional risk of GA increasing the likelihood of desaturations [12]. Furthermore, several observational studies have demonstrated the need to move away from the ongoing practice of only sampling one to two lobes in children with either PBB [7] or CF [5,6,8]. Yet, there remains a paucity of evidence for the safety and potential side effects of performing FB-BAL on all six lobes under GA for paediatric clinicians to trust this practice.

To date, hypothermia has not been addressed as a complication in paediatric bronchoscopy studies, despite established evidence that hypothermia increases the risk of infections and post-operative complications in children [9,10]. Given the short duration of our bronchoscopy procedures, theatre warming methods and core temperatures are not traditionally used, and our patients have a hospital blanket during their procedure, and remain fully dressed in the attire they arrived in. The measurements from tympanic thermometers remain highly user-dependent and are not as accurate as core thermometers. Since cold environments are known to contribute to paediatric hypothermia [9,10], we also measured our procedural room temperatures through our engineering department, and although found to be generally on the higher side above 22 °C, there was great variation from week to week, with the lowest recorded temperature at 20.9 °C and the highest at 25.04 °C. Hypothermia during paediatric bronchoscopy remains multifactorial and further studies are needed to determine the optimal room temperature and warming methods.

This study has design limitations as a single-centre observational study. Limitations include lack of statistical analysis and external validity, the potential for systematic bias and that the results may not be generalisable to other trusts depending on their policies and practices. Limitations also included reliance on clinician documentation; hence the reason cough is not listed as one of the main causes of our delayed discharges or readmissions. Additionally, procedural times were affected if the patient was electively admitted for a long-line placement for IV antibiotic treatment following their bronchoscopy, extending the time spent under GA. However, none of our patients required admission to PICU following their procedures or had severe iatrogenic complications such as pneumothorax.

## 5. Conclusions

In conclusion, paediatric FB with six-lobe BAL under GA is a safe procedure with low incidence of major complications when performed by expert clinicians. Parents should be advised of a 9% risk of delayed overnight discharge.

## Figures and Tables

**Table 1:** Indications for flexible bronchoscopy.

Prospective and Retrospective (n = 622)	n	%
Protracted bacterial bronchitis	328	52.7
Cystic fibrosis exacerbation or culture review	128	20.6
Recurrent LRTIs ^1^ or Pneumonia	41	6.6
Recurrent croup, stridor, or suspected malacia	37	6.1
Difficult asthma	30	4.8
CXR ^2^ or CT ^3^ changes, or lobar collapse	26	4.2
Non-CF bronchiectasis	17	2.7
Recurrent haemoptysis	4	0.6
Tuberculosis	4	0.6
Aspirational lung disease or recurrent aspirations	4	0.6
Primary ciliary dyskinesia	3	0.5

^1^ LRTI, lower respiratory tract infection; ^2^ CXR, chest X-ray; ^3^ CT, computed tomography.

**Table 2:** Intra- and immediate post-procedure complications.

Prospective Group (n = 120)	n	%
No complications	105	87.5
Major	4	
Significant desaturation	4	3.3
with laryngospasm ^1^	1	0.8
with coughing ^1^	1	0.8
Pneumothorax	0	0.0
Minor	11	
Transient desaturation	6	5.0
Pyrexia	1	0.8
Tachypnoea	1	0.8
Vomiting	1	0.8
Wheeze	2	1.6

^1^ One patient with significant desaturation also had laryngospasm and coughing.

**Table 3:** Major complications.

Patients (n = 4)	Indication	Age	Expected?	Complication	Outcome
P1	Probable PBB	5	No, day case procedure	Intra-op desaturation to 85% during insertion of the LMA	One day delayed discharge for pyrexia and $O_{2}$ Sats 93–94%
P2	IV antibiotics for CXR changes	5	Yes, complex elective admission	Intra-op desaturation to 77% due to large plug left main bronchus and laryngospasm	No post-procedure complications. No delay to planned elective admission
P3	Probable PBB	4	No, day case procedure	Intra-op desaturation to 88% due to cuff leak	No delayed discharge but re-admitted on the same day
P4	Probable PBB	2	Yes, slight caution	Immediate post-procedure desaturation to 87–88% due to cleft repair	One day delayed discharge for mild pyrexia and $O_{2}$ Sats 93–94%

P, patient; PBB, protracted bacterial bronchitis; Intra-op, intra-operative; IV, intravenous; CXR, chest X-ray; LMA, laryngeal mask airway; $O_{2}$ Sat, oxygen saturation.

**Table 4:** Delayed discharges.

Prospective and Retrospective (n = 622)	n	%
Pyrexia and tachypnoea requiring oxygen	15	25
Pyrexia	11	18
Due to unexpected bronchoscopy findings *	8	13
Pyrexia and tachypnoea not requiring oxygen	6	10
Transient desaturations	4	6.9
Pyrexia and vomiting	4	6.9
Significant desaturations	2	3.4
Wheeze	1	1.6
Tachycardia	1	1.6
Haemoptysis	1	1.6
No reason documented	7	12

* Findings included severe tracheomalacia and suspected vascular malformation; excess secretions with atelectasis found on CT scan; extremely friable right main bronchus and bronchus intermedius with lots of blood in the field; and thick casts of purulent material.

**Table 5:** Readmissions.

Prospective and Retrospective (n = 622)	n	%
Pyrexia	8	44.3
Pyrexia and vomiting	6	33.3
Pyrexia and tachypnoea requiring oxygen	1	5.6
Pyrexia and wheeze	1	5.6
Long-line complication	1	5.6
Viral gastroenteritis	1	5.6

## Data Availability

The data presented in this study are available on request from the corresponding author so that the publication of such data does not compromise the anonymity of the participants or breach local data protection laws due to patient personal information.
